# Attitudes toward Infection Prophylaxis in Pediatric Oncology: A Qualitative Approach

**DOI:** 10.1371/journal.pone.0047815

**Published:** 2012-10-24

**Authors:** Caroline Diorio, Deborah Tomlinson, Katherine M. Boydell, Dean A. Regier, Marie-Chantal Ethier, Amanda Alli, Sarah Alexander, Adam Gassas, Jonathan Taylor, Charis Kellow, Denise Mills, Lillian Sung

**Affiliations:** 1 Program in Child Health Evaluative Sciences, The Hospital for Sick Children, Toronto, Ontario, Canada; 2 Community Health Systems Resource Group, The Hospital for Sick Children, Toronto, Ontario, Canada; 3 Department of Haematology/Oncology, The Hospital for Sick Children, Toronto, Ontario, Canada; 4 Canadian Centre for Applied Research in Cancer Control, British Columbia Cancer Agency, Vancouver, British Columbia, Canada; 5 Program in Health Research Methodology, McMaster University, Hamilton, Ontario, Canada; University of Massachusetts Medical School, United States of America

## Abstract

**Background:**

The risks and benefits of infection prophylaxis are uncertain in children with cancer and thus, preferences should be considered in decision making. The purpose of this report was to describe the attitudes of parents, children and healthcare professionals to infection prophylaxis in pediatric oncology.

**Methods:**

The study was completed in three phases: 1) An initial qualitative pilot to identify the main attributes influencing the decision to use infection prophylaxis, which were then incorporated into a discrete choice experiment; 2) A think aloud during the discrete choice experiment in which preferences for infection prophylaxis were elicited quantitatively; and 3) In-depth follow up interviews. Interviews were recorded verbatim and analyzed using an iterative, thematic analysis. Final themes were selected using a consensus approach.

**Results:**

A total of 35 parents, 22 children and 28 healthcare professionals participated. All three groups suggested that the most important factor influencing their decision making was the effect of prophylaxis on reducing the chance of death. Themes of importance to the three groups included antimicrobial resistance, side effects of medications, the financial impact of outpatient prophylaxis and the route and schedule of administration.

**Conclusion:**

Effect of prophylaxis on risk of death was a key factor in decision making. Other identified factors were antimicrobial resistance, side effects of medication, financial impact and administration details. Better understanding of factors driving decision making for infection prophylaxis will help facilitate future implementation of prophylactic regiments.

## Introduction

Infections in pediatric oncology patients are associated with morbidity and mortality. [1] Antimicrobial agents can be used to treat documented infections, as empiric therapy for suspected infections, or as prophylaxis to prevent infections. Standard practice at most pediatric oncology centres includes aggressive empiric and treatment strategies. [2] Prophylactic strategies are less commonly used. [2].

In adults, infection prophylaxis may significantly reduce morbidity and mortality for both bacterial and fungal infections in high risk populations. [3], [4] However, even among adult patients, there are questions about the consequences of prophylaxis and the impact on antimicrobial resistance is uncertain. In pediatrics, the evidence for infection prophylaxis is much less robust. [5], [6] Further, the consequences of infection prophylaxis in young children are also uncertain.

Because of the uncertainty about the balance of risks and benefits of infection prophylaxis, preferences should be considered in the decision-making process. [7] Little is known about the attitudes of parents, children or healthcare professionals (HCPs) toward infection prophylaxis in pediatric oncology. The objective of this study was to describe the attitudes of these key stakeholders using qualitative methodology. Traditional interviews with open-ended questions and a “think aloud” (TAL) technique during a simulated decision task were employed.

## Methods

This analysis is a companion to a second study, which was designed to quantify preferences for antibiotic and antifungal prophylaxis using a discrete choice experiment (DCE) (see companion paper).

### Ethics Statement

This study was approved by The Hospital for Sick Children’s (SickKids’) Research Ethics Board. All participants consented to participation in writing. For the child respondent group, only children who were of the mental capacity to consent for themselves were approached and therefore, no consent was obtained from next of kin, caretakers or guardians on behalf of these children. Demographic information was obtained from each participant. Additional information about the child's diagnosis and treatment were abstracted from the child's chart.

### Sample

Respondents were parents of children (aged 0 to 18 years) receiving chemotherapy or hematopoietic stem cell transplant (HSCT) for cancer, children (aged 12 to 18 years) receiving chemotherapy or HSCT, or HCPs caring for pediatric oncology patients at SickKids, Toronto, Canada. One parent from each family was included. Eligible HCPs were physicians, pharmacists, social workers and nurse practitioners. Participants who could not read English were excluded.

### Study Procedures

This study occurred in three phases: (1) A qualitative pilot; (2) A TAL during a DCE; and (3) An in-depth qualitative follow-up. One investigator with experience in qualitative methodology (CD) interviewed respondents in all three phases. Two other trained investigators also interviewed respondents in phase 2. Standardized scripts were used throughout.

The qualitative pilot was conducted to identify the main 4 or 5 attributes to be used in the subsequent DCE. Interviews used a semi-structured format with open-ended questions.

For the second phase, a TAL during a DCE was used. TAL is an approach to understanding respondents’ choices in the context of stated preference methods. [8] Participants were presented with a hypothetical decision-making task using a DCE. Participants were given basic information about infections and prophylaxis. The DCE illustrated five attributes: route of administration, chance of death, chance of infection, common side effects and the cost for 28 days of treatment, with each attribute having three or four levels. For example, “chance of infection” levels for the bacterial DCE were 10%, 30%, 50% and 70%. Costs were stated to be out-of-pocket and values were derived from formulary costs for medications commonly used for prophylaxis. Participants were asked to choose between three unlabelled options: two treatment options and an opt-out option (Medication A, Medication B and No Medication). Sixteen scenarios were presented sequentially on flash cards. For the TAL, participants were asked to verbalize their thought process during decision making, and were continuously prompted to think aloud. Participants were encouraged to share relevant experiences and the interviewer asked for clarification when necessary.

In the third phase, a semi-structured interview was conducted; the interview guide was developed using data generated from phases 1 and 2. These interviews were used to clarify themes derived from the TAL analysis and to further explore aspects of decision making that had not been fully elucidated in the earlier phases of the study.

All three phases were digitally recorded and transcribed verbatim. The maximum sample sizes were decided *a priori* and interviews were continued until saturation was achieved over all three phases of the study. For phase 1, it was anticipated that 10–15 participants would be needed to identify the main 4 or 5 attributes that would be used for the DCE. For phase 2, 30 participants per respondent type in the DCE were asked to consent to audiotaping and those who consented were included. For phase 3, up to 8 participants per respondent type were targeted. [9].

### Analysis

All interview transcriptions were checked against original recordings and compared to field notes for consistency. Two authors (CD and DT) independently coded the comments, identified themes using thematic analysis and elucidated sub-themes within each theme following prolonged engagement with the transcripts. [10], [11] Sample quotes were identified to support themes and sub-themes. The study team (DT, CD, LS and KB) met repeatedly to redefine themes and sub-themes in an iterative, continuous process. Themes were further refined by checking understanding with participants.

Demographic data were analyzed using SPSS 12.

## Results

Five parents, two children and four HCPs participated in the initial open-ended interview phase. Twenty-six parents, 19 children and 21 HCPs participated in the TAL. A further four parents, one child and three HCPs participated in the open ended follow-up interview. Demographic data are presented in Table 1 and parent characteristics are similar to those who participated in the main DCE study (see companion paper).

**Table 1:** Demographic data for respondents participating in one of the three phases of the study.

Characteristic	Value
**Parent Respondents (N = 33)**	
*Respondent Characteristics*	
Median age in years (range)^a^	42 (33–54)
Male (%)	9 (27.3)
Married (%)	29 (87.9)
Median age of child in years (range)	8 (2–17)
*Child Characteristics*	
Male child (%)	21 (63.6)
Cancer type (%)	
Brain Tumor	2 (6.1)
Leukemia and other hematological malignancies	13 (39.4)
Lymphoma	5 (15.2)
Solid Tumor	12 (36.4)
Other^b^	1 (3.0)
**Child Respondents (N = 21)**	
Male (%)	11 (52.4)
Median age in years (range)	15 (12–17)
Cancer type (%)	
Leukemia and other hematological malignancies	5 (23.8)
Lymphoma	7 (33.3)
Solid Tumor	5 (23.8)
Other	4 (19.0)
**Healthcare Professional Respondents (N = 28)**	
Male (%)	11 (39.3)
Median years of experience (range)	11 (1–27)
Profession type	
Staff Physician	8 (28.6)
Oncology Fellow	9 (32.1)
Social Worker	2 (7.1)
Nurse Practitioner	6 (21.4)
Pharmacist	3 (10.7)

a missing n = 8.

### Overview

Parents, children and HCPs generally were in favor of prophylaxis. During the TAL phase, all three groups expressed that the chance of death and the chance of infection were the most important drivers of their decision making:

See that for me is the clincher. Death for me, since it’s a final thing. (Father of a 7 year old with brain tumor).

**Figure 1:**
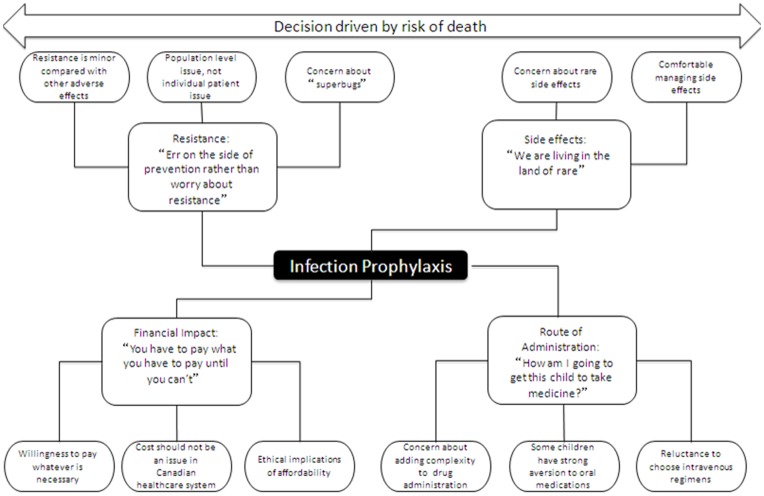
Themes and sub-themes related to infection prophylaxis from the perspective of parents, children and healthcare professionals.

Despite expressing a desire for effective prophylaxis, all three groups verbalized concerns about aspects of prophylaxis. Major issues that were identified during interviews were antimicrobial resistance, financial burden, route of administration and the impact of adverse effects of medication. Figure 1 illustrates an overview of themes and sub-themes.

### Resistance: Err on the Side of Prevention Rather than Worry about Resistance

The consensus regarding resistance was that while it was an important issue, it did not have an impact on actual individual patient decision making about infection prophylaxis. Many parents and HCPs viewed antibiotic resistance as an issue at the community level as opposed to the individual level:

When the boys were healthy there was a concern. I understood and was careful about antibiotics but now because he is not as healthy I am more selfish about that type of thing now. I think I would make that decision based on what is best for him not what is best for everyone. (Mother of a 5 year old with rhabdomyosarcoma).

HCPs also indicated that the choice of antibiotic and the choice of population for prophylaxis were important considerations in the context of resistance:

From a population perspective I am treating a small fraction of the kids that come through here, so my use of antibiotics in that setting would not be anticipated to have any impact on community health… so it is not that antibiotic resistance is irrelevant but it is not the be all and end all for my type of practice. (Physician).

While confirming their desire for preventative medications, parents expressed concern about the potential of developing “super bugs” after taking antibiotics:

It would be something I would think about. It would not be the driving thing but I would think, maybe could this create a super bug? (Father of a 7 year old with brain tumor).

### Financial Burden: You have to Pay what you have to Pay Until you can’t

Participants found weighing the cost of a medication versus potential benefit difficult. A common theme expressed was a willingness to do whatever was necessary to provide beneficial care. HCPs acknowledged that families will do whatever they are asked if they think it will help their child:

You have to pay what you have to pay until you can’t. That’s why they invented bankruptcy. (Father of a 4 year old with neuroblastoma).

Children expressed an understanding of both the necessity of paying for medication and the impact of the cost of medication on their family:

I know that we would probably be willing to pay as much as we would need to … But we know how to budget our money and we know how to cut back sometimes, we have had to do that sometimes, so I do not think it would hit our family too hard and as long as it would help me and benefit me then we would probably get it. (14 year old with histiocytosis).

Some HCPs felt that the cost of medication should be irrelevant to decision making for infection prophylaxis, because in their experience, costs of medication can be covered through special access grants and that cost should not be an issue in the Canadian healthcare system:

I do not really look at that. Okay, it is expensive, fine but if that is what the patient needs, that is what the patient needs … I guess I’m tempted to not look at that at all. (Physician)

In spite of the cost. I don’t care about the cost. (Physician)

Other HCPs expressed concern about adding an additional obligation to the already heavy financial burden that families carry:

If you pick a number, $200 a month, for a family that has significantly low income. That $200 might impact other things that could equally be as detrimental to health, the food that they buy or, they are going to cut on something else which would be equally as important. (Pharmacist)

Some HCPs expressed concern about the ethical implications of implementing infection prophylaxis if it would be unaffordable to some families:

Ethically it’s such a morally distressing situation … you’re saying we have this that we hope could decrease the chances but there’s no coverage… if this is something to possibly introduce, how would we balance out the families that have all those resources… and then the families that just don’t have that? (Social worker)

### Route of Administration: How am I Going to Get this Child to Take Medicine?

Adding an additional medication to already complicated treatment regimens was a matter of careful consideration for all three groups interviewed.

Many parents articulated a real concern about giving additional oral medication to their children. Parents described difficulty with oral medication administration:

I think most kids hate pills. Any kind of oral medication. And it’s largely psychological. (Father of a 10 year old with Ewing’s sarcoma)

He has such a hard time with pills. If he could take it once per day instead of twice per day it is better. Better for him. (Mother of a 13 year old with lymphoma)

Similarly, children tended to prefer to not take oral medication and to take as little medication as possible:

This is a lot of medication. So already I am turned off. (16 year old with leukemia)

In contrast, HCPs expressed a preference for oral regimens:

But obviously an oral route is preferable for a prophylactic regimen. But also the age of the child comes in to the equation. Sometimes it’s hard to give oral medications to very young children. (Physician)

### Side Effects: We are Living in the Land of Rare

Contrary to *a priori* expectations, participants felt comfortable managing the potential side effects of medication. Several parents expressed concern about rare side effects:

It’s just listing you know like the most common of the side effects … my [child] has two rare diseases. … So I would like to know the rare side effects. (Mother of a 12 year old with leukemia)

HCPs expressed concern about side effects in the context of compliance but were also confident in their ability to manage both minor and serious side effects of medication:

There are different aspects that play into account when you have a high amount of side effects. It is difficult to get them to take medication 2 times a day if they are really suffering…However you deal with side effects in oncology all the time and there is medication for that as well. (Physician)

## Discussion

We found that in general, parents, children and HCPs were in favor of infection prophylaxis. The results suggest that the most important issue driving decision making was effect of prophylaxis in reducing the risk of death. Other important issues identified were antibiotic resistance, financial burden, side effects and route of administration.

To our knowledge, this is the first study to use qualitative methodology to describe parent, child and HCP preferences for infection prophylaxis in the context of pediatric oncology. Our findings suggest that financial burden and route of administration in particular are contentious issues.

We found that all respondent groups were concerned about costs of prophylaxis. Our study presented the costs of medication based on formulary values of potential prophylactic medications; HCPs and parents may be unaware of the full cost of these medications. Our study suggests that if prophylaxis is implemented in a pediatric oncology program, that costs of the regimen and coverage of costs should be explicitly considered as parents are likely to find a way to pay for these medications even if it means cutting back on other important services or goods.

Route of administration was an important issue to all groups, particularly parents. Many parents of children of all ages expressed concern about having their child take oral medications. The concerns about oral administration may suggest that adherence could be problematic, a concern recognized by HCPs. HCPs were wary of prescribing parenteral prophylaxis because they felt it would be logistically impractical outside of the hospital setting. Minimizing the number of oral administrations is likely to be an important component of successful prophylaxis programs.

One limitation of our study is that participants were recruited at a single center. Attitudes toward prophylaxis, particularly among HCPs, may differ at other centers and in other countries. Second, our study explored attitudes toward a hypothetical supportive care regimen. Opinions of all parties surveyed may change if the regimen was implemented. Third, we chose an approach where we continued to interview participants until saturation was achieved over all three phases of the study. This approach limited the number of participants for the third phase of our study and consequently, it is possible that some themes were missed.

Infectious morbidity and mortality remain important entities in pediatric oncology, particularly in high risk populations. Because infection prophylaxis would require long term adherence to an antibiotic regimen, the attitudes and beliefs of stakeholders are an important consideration. Our findings may facilitate future implementation of prophylactic programs. A better understanding of stakeholder concerns may help facilitate patient centered collaboration between HCPs, patients and families.

## References

[1] Sung L, Lange BJ, Gerbing RB, Alonzo TA, Feusner J (2007) Microbiologically documented infections and infection-related mortality in children with acute myeloid leukemia. Blood 110: 3532–3539. 17660380 10.1182/blood-2007-05-091942

[2] Lehrnbecher T, Ethier MC, Zaoutis T, Creutzig U, Gamis A, et al (2009) International variations in infection supportive care practices for paediatric patients with acute myeloid leukaemia. Br J Haematol 147: 125–128. 19663826 10.1111/j.1365-2141.2009.07844.x

[3] Gafter-Gvili A, Fraser A, Paul M, Leibovici L (2005) Meta-analysis: antibiotic prophylaxis reduces mortality in neutropenic patients. Ann Intern Med 142: 979–995. 15968013 10.7326/0003-4819-142-12_part_1-200506210-00008

[4] Robenshtok E, Gafter-Gvili A, Goldberg E, Weinberger M, Yeshurun M, et al (2007) Antifungal prophylaxis in cancer patients after chemotherapy or hematopoietic stem-cell transplantation: systematic review and meta-analysis. J Clin Oncol 25(34): 5471–5489. 17909198 10.1200/JCO.2007.12.3851

[5] Alexander S, Nieder M, Zerr DM, Fisher BT, Dvorak CC, et al (2011) Prevention of bacterial infection in pediatric oncology: What do we know, what can we learn? Pediatr Blood Cancer 59: 16–20. 22102612 10.1002/pbc.23416 PMC4008322

[6] Dvorak CC, Fisher BT, Sung L, Steinbach WJ, Nieder M, et al (2012) Antifungal prophylaxis in pediatric hematology/oncology: New choices & new data. Pediatr Blood Cancer 59: 21–26. 22102607 10.1002/pbc.23415 PMC4008331

[7] Guyatt G, Gutterman D, Baumann MH, Addrizzo-Harris D, Hylek EM, et al (2006) Grading strength of recommendations and quality of evidence in clinical guidelines: report from an american college of chest physicians task force. Chest 129: 174–181. 16424429 10.1378/chest.129.1.174

[8] Ryan M, Watson V, Entwistle V (2009) Rationalising the 'irrational': a think aloud study of discrete choice experiment responses. Health Economics 18: 321–336. 18651601 10.1002/hec.1369

[9] McCracken G (1989) The Long interview. Palo Alto, CA: Sage Publications.

[10] Tuckett AG, Tuckett AG (2005) Applying thematic analysis theory to practice: a researcher's experience. Contemporary Nurse 19: 75–87. 16167437 10.5172/conu.19.1-2.75

[11] Braun V, Clarke V (2006) Using thematic analysis in psychology. Qual Res Psychol 3: 77–101.

